# Metagenomics Analysis of Microorganisms in Freshwater Lakes of the Amazon Basin

**DOI:** 10.1128/genomeA.01440-16

**Published:** 2016-12-22

**Authors:** Danyelle Toyama, Luciano Takeshi Kishi, Célio Dias Santos-Júnior, Andrea Soares-Costa, Tereza Cristina Souza de Oliveira, Fernando Pellon de Miranda, Flávio Henrique-Silva

**Affiliations:** a Department of Genetics and Evolution, Center for Biological and Health Sciences, Federal University of São Carlos, São Carlos, São Paulo, Brazil; b Department of Chemistry, Federal University of Amazonas, Manaus, Amazonas, Brazil; c Petróleo Brasileiro S.A. (Petrobras), Centro de Pesquisas e Desenvolvimento Leopoldo Américo Miguez de Mello, Rio de Janeiro, Brasil

## Abstract

The Amazon Basin is the largest hydrographic basin on the planet, and the dynamics of its aquatic microorganisms strongly impact global biogeochemical cycles. However, it remains poorly studied. This metagenome project was performed to obtain a snapshot of prokaryotic microbiota from four important lakes in the Amazon Basin.

## GENOME ANNOUNCEMENT

The Solimões-Amazon River drains the Andes Mountains by crossing a humid and flat region to the Atlantic Ocean. This complex fluvial system carries 1,240 megatons of sediments per year and reworks approximately 3,200 megatons of floodplain sediments per year (1). Its annual discharge of 6.3 trillion m^3^ represents approximately 16% of all freshwater released into the world’s oceans (2).

The Amazon region includes a large number of rivers and lakes, as well as the world’s largest rainforest characterized by enormous biodiversity. However, the microbiome of this habitat has been poorly studied. In Brazil, the first freshwater metagenomics study performed in the Solimões River revealed a diverse community with a vast array of potential metabolisms in the largest hydrographic basin on the planet (3).

Freshwater environments and their microbial communities configure the basis of the food web and are the major agents of biogeochemical cycling (4). These environments differ from soil, marine, and freshwater environments in their composition of microbial communities. Temperature, pH, hydrological retention time, and trophic state can determine the composition of microbial communities of lakes (5, 6). Some lakes may receive water from nearby rivers in the flood season, which can change the microbial community of these environments. In the Amazon region, these lakes receive material from the Solimões River, for example. In this work, we performed metagenomics high-throughput sequencing to understand the ecology of microbial communities from lakes adjacent to the Solimões River.

We collected water samples in September 2008 (dry season) from Lake Poraquê (03º57’36.36″S; 63°09′48.17″W), Lake Preto (03º21’12.46″S; 60°37′31.30″W), Manacapuru Great Lake (03º15’50.96″S; 60°41′19.76″W), and Lake Ananá (03º53’11.83″S; 61°40′36.75″W). Sampling and total DNA extraction were conducted using a methodology described elsewhere (3). We sequenced metagenomic DNA with paired-end 2 × 100-bp reads on an Illumina HiSeq 2500 platform (Illumina, Inc., San Diego, CA, USA). Reads were then subjected to quality trimming using the NGS QC toolkit version 2.3.3 software (7) by discarding low-quality data (<Q20). A total of 334 million sequences for Lake Preto, 349 million sequences for Manacapuru Great Lake, 376 million sequences for Lake Ananá, and 165 million sequences for Lake Poraquê were submitted for annotation and classification using the MG-RAST server (8).

Taxonomic assignment revealed that metagenomes contained 89 to 98% (of assigned reads) bacteria, 0.3 to 1.2% archaea, and 0.5 to 2.5% viruses. The major observed bacterial phyla included *Proteobacteria* (abundance percentages: 36.1 to 59.5%), *Cyanobacteria* (1.8 to 32.6%), *Actinobacteria* (12.7 to 28.2%), Planctomycetes (0.5 to 3.6%), Bacteroidetes (1.9 to 3.5%) and Firmicutes (2.3 to 5.5%). Functional annotations revealed a considerable fraction of reads associated with respiration (3.3 to 3.8%), photosynthesis (0.2 to 1.1%), and phages, prophages, transposable elements, and plasmids (1.6 to 3.6%). This metagenome project provides valuable information for future studies about the metabolic potential of microbial communities present in lakes in this region.

### Accession number(s).

The sequences obtained in this project have been deposited in the NCBI Short Read Archive under the following GenBank accession numbers: SRX659579 (Lake Poraquê), SRX659576 (Lake Preto), SRX659577 (Manacapuru Great Lake), and SRX659578 (Lake Ananá).
